# Aplastic Anemia Proceeding to Thymoma: A Rare Co‐Occurrence—A Case Report and Review Article

**DOI:** 10.1155/crh/6971236

**Published:** 2026-04-01

**Authors:** Maryam Ghazizadeh, Matin Ghazizadeh, Mohammad Moini

**Affiliations:** ^1^ Department of Hematology-Oncology, Shahid Modarres Hospital, Shahid Beheshti University of Medical Sciences, Tehran, Iran, sbmu.ac.ir; ^2^ Department of Otolaryngology, Head and Neck Surgery, Taleghani Hospital, Shahid Beheshti University of Medical Sciences, Tehran, Iran, sbmu.ac.ir; ^3^ School of Medicine, Tehran University of Medical Sciences, Tehran, Iran, tums.ac.ir

**Keywords:** anemia, aplastic, autoimmune disease, immunohematology, thymoma

## Abstract

**Background:**

Malignant thymoma is a rare tumor associated with various paraneoplastic syndromes, such as myasthenia gravis, pure red cell aplasia, and hypogammaglobulinemia. Aplastic anemia (AA) is an uncommon complication of thymoma that may occur during the disease or after thymoma resection. We report a case presented with AA before the thymoma.

**Case:**

We report the case of a 34‐year‐old man with thymoma who developed AA before the detection of the tumor. Although pancytopenia did not improve after thymectomy, prolonged immunosuppressive therapy led to complete resolution of AA. The patient has no recurrence during a 42‐month follow‐up period.

**Conclusion:**

AA may precede the detection of thymoma and represent its initial clinical manifestation. So, thymoma should be considered a rare cause, along with other more common etiologies of AA. Patients with AA and thymoma require prolonged immunosuppressive therapy after surgery.

## 1. Introduction

Malignant thymoma is a rare tumor associated with various paraneoplastic syndromes, such as myasthenia gravis (MG), hypogammaglobulinemia, and autoimmune hematologic diseases [1]. Pure red cell aplasia (PRCA) accounts for the most common associated hematologic features and occurs in 2%–5% of cases. Aplastic anemia (AA) is an uncommon complication of thymoma and may develop after tumor resection. The co‐occurrence of AA and thymoma is estimated at 0%–1.4% [2]. It is an autoimmune manifestation of thymoma that may occur during the course of the disease or after thymoma resection. Immunosuppressive therapy is recommended for patients with thymoma and AA. In refractory cases, allogeneic hematopoietic stem cell transplantation (HSCT) may be needed. The authors report a case in which severe AA was the initial clinical presentation, leading to the subsequent detection of thymoma during etiologic evaluation.

## 2. Case Presentation

A 34‐year‐old man was admitted to the hospital with chief complaints of weakness, fever, night sweats, and weight loss for 40 days. He complained of a sore throat and sputum. At first, he was misdiagnosed as a COVID‐19 patient, but he tested negative for COVID‐19 PCR testing. Physical examinations revealed a perianal abscess. Initial laboratory evaluation showed pancytopenia with hemoglobin 9.9 g/dL, white blood cell count 1.6 × 10^9^/L (29% neutrophils; absolute neutrophil count 0.46 × 10^9^/L), and platelet count 117 × 10^9^/L, reticulocyte index 0.6%, a reduced CD4:CD8 ratio of 0.63, together with elevated erythrocyte sedimentation rate (ESR), C‐reactive protein (CRP), and serum ferritin. Serum blood urea nitrogen (BUN), creatinine (Cr), liver function tests (LFTs), and lactate dehydrogenase (LDH) were within normal limits. Since the patient was showing signs of fever and pancytopenia, he was checked for cytomegalovirus antibody (CMV Ab), Epstein–Barr virus antibody (EBV Ab), human immunodeficiency virus antibody (HIV Ab), and hepatitis viral markers; all appeared to be normal. Bone marrow aspiration and biopsy showed a hypocellular marrow (< 25%) with normal morphology of all three lineage hematopoietic cells; however, there was no increase in the number of blasts, and the marrow karyotype appeared normal. During an abdominopelvic computed tomography (CT) scan, splenomegaly was identified, as evidenced by the enlarged size of the spleen 157 mm. In an abdominal color Doppler ultrasound, the portal vein exhibited a peak systolic velocity (PSV) of 13 cm/s, and the flow within the splenic, portal, and hepatic veins was normal, with no indications of thrombosis. A lung CT scan detected a heterogeneous enhancing mass measuring 65 ∗ 86 mm on the left side of the anterior mediastinum. A mild compression of the heart, and the nearby left lung was also noted (Figure 1).

FIGURE 1The axial view of the chest CT scan without (a) and with contrast injection (b), showing a heterogenous enhancing mass measured 65 ∗ 86 mm in the left side of the anterior mediastinum.

**Figure:**
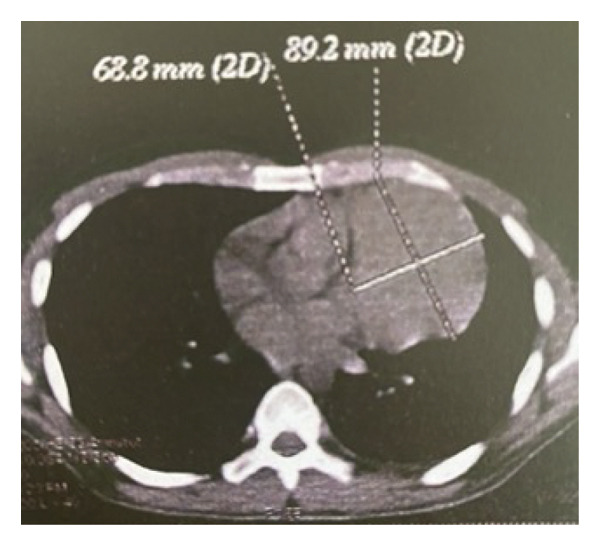
(a)

**Figure:**
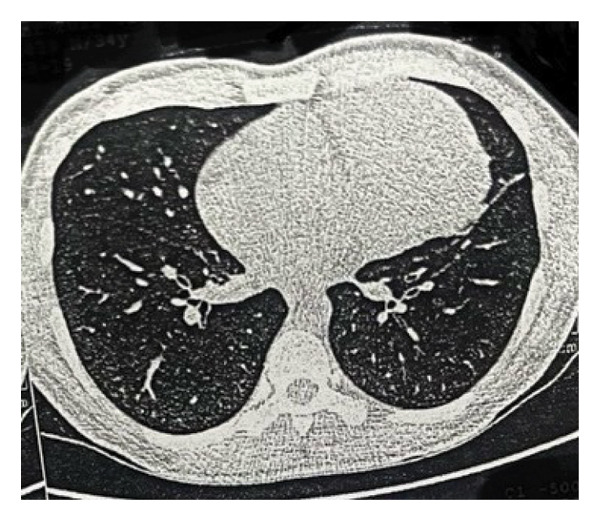
(b)

**FIGURE 2:**
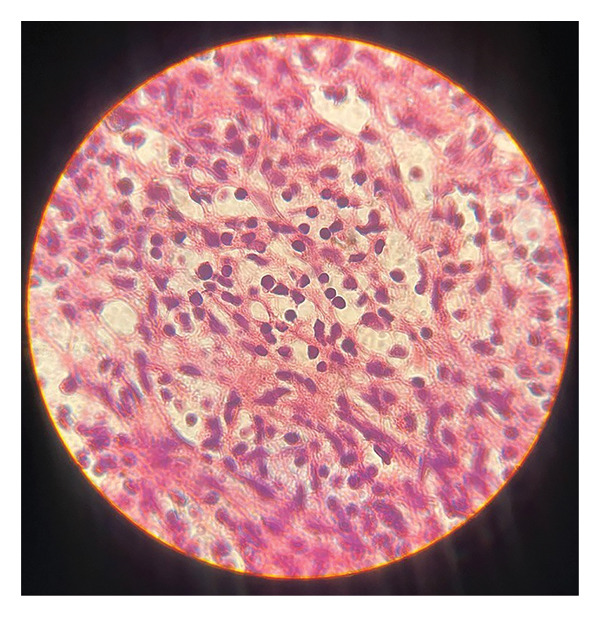
Microscopic image of resected tumor confirmed as thymoma.

The patient underwent surgery during which a total mass excision was performed via mediastinotomy. Histologic examination revealed polygonal neoplastic epithelial cells, numerous thymocytes, and a significant focus of cystic changes, compatible with thymoma (Figure 2). The diagnosis was confirmed by immunohistochemistry. According to the WHO classification, the tumor was consistent with a type B2 thymoma and was staged as Masaoka–Koga stage III. Despite tumor removal, the patient’s pancytopenia did not improve; therefore, immunosuppressive therapy was initiated. Cyclosporine was started at 5 mg/kg/day for 8 days, then reduced to 3 mg/kg/day, maintaining a trough level of 200–400 ng/mL, with dose adjustments according to LFTs. Cyclosporine was continued for 6 months, gradually tapered, and discontinued after 8 months in total. Prednisone was administered at 2 mg/kg/day for the first 14 days, followed by 1 mg/kg/day from day 15 to day 45, and then tapered weekly, reaching complete discontinuation by 8 months. Intravenous immunoglobulin (IVIG) was given once, at 20 g/day for 5 consecutive days. Pancytopenia was completely corrected 6 months after initiation of the therapy, with hemoglobin 13 g/dL, white blood cell count 4.5 × 10^9^/L (absolute neutrophil count≈2.7 × 10^9^/L), and platelet count 189 × 10^9^/L. These values are compatible with a complete hematologic response according to the British Committee for Standards in Haematology (BCSH) criteria [3]. The patient has been followed for 42 months without any signs of recurrence.

## 3. Discussion

AA is a type of bone marrow failure that results in pancytopenia and marrow hypoplasia, with increased fat and reduced blood‐forming cells in the marrow. The main mechanism of AA is immune‐mediated destruction of hematopoietic stem cells by type 1 cytotoxic T cells [4]. Thymoma is the most common mediastinal tumor and can be associated with various autoimmune syndromes, such as PRCA [5], MG, and AA. AA is a rare complication of thymoma and may not resolve after thymectomy [6]. Thymoma‐associated immune dysregulation results from aberrant maturation of T‐cell precursors, leading to the release of self‐reactive CD4/CD8 T‐cell clones, inversion of the CD4/CD8 ratio, and increased cytotoxic activity, ultimately causing immune‐mediated bone marrow suppression [7]. Therefore, removing the thymus may not affect the clinical course of AA. On the other hand, severe AA may occur several years after thymectomy without any sign of primary tumor recurrence [8, 9]. Immunosuppressive therapy is recommended for patients with AA and thymoma, and allogenic bone marrow transplantation may be needed in refractory cases. In patients with acquired AA, a thorough etiologic evaluation, including exclusion of viral infections, inherited disorders, and secondary autoimmune conditions, is essential, and imaging studies such as contrast‐enhanced chest CT should be considered to rule out thymoma when no alternative cause is identified.

Several autoimmune diseases are linked to thymoma. In the French multicenter survey of patients with thymoma‐associated AA, autoimmune diseases reported within this subgroup included MG (11.4%), immune thrombocytopenia (8.6%), PRCA (5%), coeliac disease (2.9%), and autoimmune hemolytic anemia (2.9%) [10]. AA is a rare condition that can also occur with thymoma, with a reported frequency of 1.4%. According to the same survey, the median age of patients with thymoma‐associated AA was 66 years. The severity of AA included nonsevere AA in 55% of patients, severe AA in 33%, and very severe AA in one case. In our case, based on the hypocellular bone marrow (< 25%) and peripheral blood counts (absolute neutrophil count approximately 0.46 × 10^9^/L and reticulocyte index < 1%), the disease was classified as severe AA according to the modified Camitta/Bacigalupo criteria [11, 12]. The interval between the diagnoses of AA and thymoma in the French cohort did not exceed 2.5 months, whereas in our patient, it was approximately 2 weeks. Hypogammaglobulinemia was reported in 36% of patients in the same cohort but was not present in our case. Furthermore, there was no documented B‐cell depletion or laboratory evidence of Good syndrome in our patient; the perianal abscess was most likely attributable to severe neutropenia at presentation. Thymoma remission occurred in 96.8% of cases after thymectomy; however, no hematologic responses were observed following surgery. Consistently, our patient’s AA did not improve after thymectomy. In a different study, 66% of patients with AA and thymoma responded to antithymocyte globulin (ATG) and cyclosporine, and stem cell transplantation led to complete remission in 100% of patients [13].

In another case report, an advanced thymoma showed significant size reduction after cyclosporine therapy, with no improvement in PRCA [14]. Nevertheless, thymectomy followed by long‐term immunosuppressive therapy is recommended for immunologic complications of thymoma in adult and pediatric patients [15]. Patients with refractory PRCA or metastatic thymoma may be managed by allogeneic stem cell transplantation [16]. Our case responded to cyclosporine and prednisolone, and the relatively short interval between diagnosis and initiation of immunosuppressive therapy may have contributed to the favorable hematologic outcome.

In addition to PRCA, Good syndrome, and other immune cytopenias, amegakaryocytic thrombocytopenia has also been described as a thymoma‐associated autoimmune manifestation. Chiatamone Ranieri et al. reported a heavily pretreated patient with thymoma who developed concurrent amegakaryocytic thrombocytopenia, AA, and Good syndrome [17]. Duarte Rodrigues et al. described a case in which severe AA developed after thymectomy, preceded by marked megakaryocytic depletion on bone marrow examination [8]. Furthermore, Simkins et al. reported a patient with thymoma who initially presented with acquired amegakaryocytic thrombocytopenia and PRCA, which subsequently progressed to AA and ultimately required allogeneic stem cell transplantation for hematologic recovery [16]. These reports highlight amegakaryocytic thrombocytopenia as an important but underrecognized entity within the spectrum of thymoma‐associated immune cytopenias. In contrast, our patient did not demonstrate isolated megakaryocytic depletion or sequential cytopenias but rather presented with overt severe AA at initial evaluation.

To better contextualize our case, we conducted a systematic literature review of published reports on thymoma‐associated AA. We identified 19 well‐documented cases in the literature, including individual case reports and case series, from which we extracted the following clinical variables: patient age and sex; thymoma histology and Masaoka–Koga stage; associated autoimmune conditions; timing of AA; treatments administered; and hematologic and oncologic outcomes [7, 8, 13–29]. (Table 1)

**TABLE 1:** Summary of reported cases of thymoma‐associated aplastic anemia.

Author (year)	Age/sex	WHO subtype	Masaoka stage	Autoimmune conditions	AA timing	AA treatment	Hematologic response	Thymoma treatment	Oncologic outcome
Kobayashi (1993)	64/F	NR	NR	ITP	4 yrs postop	Cyclosporine, G‐CSF, splenectomy	Complete	Thymectomy	NR
De Giacomo (1995)	43/F	NR	I	None	Concurrent	Cyclosporine, prednisone	Complete	Thymectomy	No recurrence at 30 months
Liozon (1998)	65/M	NR	NR	None	Concurrent	Cyclosporine A	Partial	Thymectomy	NR
Dinçol (2000)	38/M	NR	NR	None	3 months postop	ALG, cyclosporine, G‐CSF, prednisone	Complete	Thymectomy	NR
Ritchie (2002)	50/M	NR	NR	MG	9 months posteradication	ATG, cyclosporine, prednisone, G‐CSF	Partial	Surgery + chemotherapy + radiotherapy	No recurrence
Park (2003)	60/F	NR	NR	None	16 months postop	Cyclosporine, G‐CSF	Complete	Thymectomy	No recurrence
Dragani (2020)	62/M	NR	NR	PRCA, AAMT	Progression after chemotherapy	Cyclosporine, prednisone, eltrombopag, ATG	None	Chemotherapy (CAPP regimen)	Progressive then stable disease
Gaglia (2007)	75/F	NR	NR	None	7 yrs postdiagnosis	Cyclosporine	Complete	Chemotherapy	Death due to progression
Nakamura (2009)	74/F	B1	I	MG	Concurrent	Transfusions	None	Extended thymothymectomy	NR
Dvir (2019)	68/M	NR	NR	None	8 yrs postdiagnosis	prednisone, cyclosporine, eltrombopag, ATG	None	None	Death
Franchi (2020)	60/F	A	I	MG	Postop	G‐CSF, cyclosporine, ATG	None	Thymectomy	No recurrence
Hayashida (2022)	71/F	B3	III	None	11 years after thymoma diagnosis	ATG, prednisone, cyclosporine, eltrombopag, romiplostim	Partial	Thymectomy, repeat resections	Tumor reduction, stable 7 months
Sun (2026)	47/F	B1	NR	MG	2 weeks postop	Allo‐HSCT, cyclosporine, mycophenolate mofetil, methotrexate	Complete	Thymectomy	NR
Simkins (2019)	61/F	NR	NR	PRCA, AAMT	Postthymectomy progression	Cyclosporine, prednisone, ATG, allo‐SCT	Complete	Chemotherapy, thymectomy	No recurrence
Chiatamone Ranieri (2019)	53/M	B2/B3	IVb	Good syndrome, PRCA, AATP	Progression after long‐standing thymoma	IVIG, prednisone, eltrombopag, ATG	Partial	Surgery, chemotherapy, radiotherapy	Stable metastatic disease
Muratori (2020)	60/M	NR	IV	PRCA	Postchemotherapy progression	Cyclosporine, prednisone, eltrombopag, cyclophosphamide	None	ADOC chemotherapy	Tumor reduction
Toret (2018)	14/M	A	I	None	Concurrent	ATG, cyclosporine, methylprednisolone, prednisolone	Complete	Thymectomy	NR
Duarte (2020)	53/M	B1	NR	AAMT	3 years postop	Cyclosporine, prednisone, Allo‐HSCT	None	Thymectomy	No recurrence
Trisal (2007)	44/M	A	NR	ITP	Concurrent	Cyclosporine, G‐CSF, Allo‐HSCT	Complete	Thymectomy	No recurrence

*Note:* AAMT = acquired amegakaryocytic thrombocytopenia; ALG = antilymphocyte globulin; ATG = antithymocyte globulin; CAPP = cyclophosphamide, doxorubicin, cisplatin, prednisone; ADOC = doxorubicin (Adriamycin), cisplatin, vincristine (Oncovin), cyclophosphamide; IVIG = intravenous immunoglobulin; yrs = years; op = operation.

Abbreviations: AA = aplastic anemia, AATP = acquired amegakaryocytic thrombocytopenic purpura, Allo‐HSCT = allogeneic hematopoietic stem cell transplantation, Allo‐SCT = allogeneic stem cell transplantation, G‐CSF = granulocyte colony‐stimulating factor, ITP = immune thrombocytopenic purpura, MG = myasthenia gravis, NR = not reported, PRCA = pure red cell aplasia, WHO = World Health Organization.

Most patients were middle‐aged or elderly, although pediatric cases have been reported. Histologically, a range of WHO subtypes was observed, including type A, B1, B2/B3, and B3 thymomas, with many cases lacking specific classification. Several patients exhibited associated autoimmune syndromes such as PRCA, immune thrombocytopenic purpura (ITP), Good syndrome, MG, or amegakaryocytic thrombocytopenia. While some patients presented with AA concurrently with thymoma or shortly after resection, others developed it years later, reinforcing the concept that thymoma may exert long‐term immune‐mediated effects. Thymectomy alone was rarely sufficient for hematologic remission; immunosuppressive therapies, particularly cyclosporine with or without ATG, constituted the mainstay of treatment. A minority of patients required HSCT. Mortality occurred in a limited number of cases and was variably attributed to disease progression or treatment‐related complications.

Our case aligns with the less frequent phenotype in which AA precedes thymoma detection. The absence of overlapping autoimmune cytopenias or hypogammaglobulinemia distinguishes it from several prior reports. Furthermore, the complete hematologic recovery achieved with immunosuppressive therapy (cyclosporine, corticosteroids, IVIG) without HSCT emphasizes the potential for nontransplant approaches in carefully selected patients.

## 4. Conclusion

AA is an uncommon but clinically significant immune‐mediated association of thymoma that may precede, coincide with, or follow thymoma diagnosis and treatment. Although rare, thymoma should be considered in the differential evaluation of acquired AA, particularly in patients with concomitant or evolving autoimmune manifestations. Recognition of this association is important, as immunosuppressive therapy remains the cornerstone of management and may achieve durable hematologic remission without the need for transplantation in selected cases.

## Author Contributions

Maryam Ghazizadeh: patient evaluation and treatment and clinical management.

Matin Ghazizadeh: data gathering, manuscript review, and supervision.

Mohammad Moini: literature review, manuscript drafting, and coordinated revisions.

Mohammad Moini had full access to all of the data in this study and takes complete responsibility for the integrity of the data and the accuracy of the data analysis.

## Funding

The authors have nothing to report.

## Disclosure

All authors have read and approved the final version of the manuscript.

## Ethics Statement

The study was approved by the ethics committee of Shahid Beheshti University of Medical Sciences.

## Consent

Informed consent was obtained from the patient to publish this report in accordance with the journal’s patient consent policy.

## Conflicts of Interest

The authors declare no conflicts of interest.

## Data Availability

The data that support the findings of this study are available on request from the corresponding author. The data are not publicly available due to privacy or ethical restrictions.
